# Rapid and Precise Measurement of Serum Branched-Chain and Aromatic Amino Acids by Isotope Dilution Liquid Chromatography Tandem Mass Spectrometry

**DOI:** 10.1371/journal.pone.0081144

**Published:** 2013-12-05

**Authors:** Ruiyue Yang, Jun Dong, Hanbang Guo, Hongxia Li, Shu Wang, Haijian Zhao, Weiyan Zhou, Songlin Yu, Mo Wang, Wenxiang Chen

**Affiliations:** 1 The Key Laboratory of Geriatrics, Beijing Hospital and Beijing Institute of Geriatrics, Ministry of Health, Beijing, China; 2 Beijing Hospital and National Center for Clinical Laboratories, Ministry of Health, Beijing, China; University of Edinburgh, United Kingdom

## Abstract

**Background:**

Serum branched-chain and aromatic amino acids (BCAAs and AAAs) have emerged as predictors for the future development of diabetes and may aid in diabetes risk assessment. However, the current methods for the analysis of such amino acids in biological samples are time consuming.

**Methods:**

An isotope dilution liquid chromatography tandem mass spectrometry (ID-LC/MS/MS) method for serum BCAAs and AAAs was developed. The serum was mixed with isotope-labeled BCAA and AAA internal standards and the amino acids were extracted with acetonitrile, followed by analysis using LC/MS/MS. The LC separation was performed on a reversed-phase C_18_ column, and the MS/MS detection was performed via the positive electronic spray ionization in multiple reaction monitoring mode.

**Results:**

Specific analysis of the amino acids was achieved within 2 min. Intra-run and total *CV*s for the amino acids were less than 2% and 4%, respectively, and the analytical recoveries ranged from 99.6 to 103.6%.

**Conclusion:**

A rapid and precise method for the measurement of serum BCAAs and AAAs was developed and may serve as a quick tool for screening serum BCAAs and AAAs in studies assessing diabetes risk.

## Introduction

Diabetes has become a global epidemic that threatens public health and the economy in most countries. Given the availability of effective interventions for delaying or preventing the onset of diabetes, early identification of at-risk individuals is particularly crucial [1]. Recent studies based on metabolomics have documented the potential key role of branched-chain (BCAAs) and aromatic (AAAs) amino acids in insulin resistance and obesity [2]–[4]. Furthermore, these amino acids have emerged as predictors of the future development of diabetes and may aid in diabetes risk assessment [5], [6].

Further studies of BCAAs and AAAs as early diabetes risk factors require simple and reliable methods for measurement of the amino acids. There have been numerous methods for amino acid analysis, but most of them seem to be too time-consuming or tedious (requiring long chromatography run time and/or multi-step sample preparation, such as derivatization) for the purpose of diabetes risk screening. The traditional ion-exchange chromatography method [7] was first developed in the 1940s and has evolved into the amino acid analyzers in use today for certain applications. A long chromatography run time is characteristic of this method. Since the 1970s, various high-performance liquid chromatography methods for the analysis of amino acids have been studied [8]. In these methods, amino acids are derivatized before the chromatography analysis for detection and separation reasons. Other techniques, such as gas chromatography [9], capillary electrophoresis [10], gas chromatography mass spectrometry [11], and liquid chromatography mass spectrometry [12], [13] have also been applied to the analysis of amino acids, most of which also require precolumn derivatization. With the development of highly selective and sensitive tandem mass spectrometry techniques, the analysis of underivatized amino acids has become possible. Several liquid chromatography tandem mass spectrometry (LC/MS/MS) methods for the direct analysis of amino acids in biological samples have been reported [14]–[17]. These methods are advantageous in that there is no need for a precolumn derivatization that costs time and can often lead to analytical problems. However, current LC/MS/MS methods mostly focus on amino acid and metabolite profiling, and often require a long LC run time.

Some commercial amino acid analysis kits based on the above analytical principles have also been available. Among them, the MS/MS kit intended for use in newborn screening is high-throughput. However, this kit focuses on amino acids and metabolites that are important for the detection of inborn metabolic disorders and does not separate the isobaric leucine and isoleucine which are important BCAAs potentially predictive of diabetes risks.

In this study, we investigated the LC separation and MS/MS detection of BCAAs and AAAs, especially that of the isobaric leucine and isoleucine, and developed a specific and precise method for the measurement of serum BCAAs and AAAs. The method requires minimum sample preparation, performs with a short run time and is suitable for screening potential diabetes risks.

## Materials and Methods

### Ethics Statement

This study has been reviewed and approved by the Ethics Committee of Beijing Hospital, Ministry of Health. All studied individuals had been made aware in writing of the intended use of their sample and provided written consent.

### Chemicals and Reagents

Standards of BCAAs [valine (Val), isoleucine (Ile) and leucine (Leu)] and AAAs [tyrosine (Tyr) and phenylalanine (Phe)], HPLC-grade acetonitrile and formic acid were obtained from Sigma-Aldrich (St. Louis, MO, USA). Isotopically labeled internal standards for each of the amino acids (Val-D_8_, Ile-D_10_, Leu-D_3_, Tyr-D_4_ and Phe-D_5_), with isotopic purities of 99%, were purchased from Cambridge Isotope Laboratories (Andover, MA, USA).

### BCAAs and AAAs Measurement by ID-LC/MS/MS

#### Calibrators and internal standards

A stock standard mixture was prepared by dissolving the amino acids in 0.1% (v:v) formic acid to a concentration of 100 mg/l for each amino acid. Calibrators for Val (330.2, 165.1, 82.5, 41.3 and 20.6 µmol/l), Ile (314.7, 157.4, 78.7, 39.3 and 19.7 µmol/l), Leu (296.4, 148.2, 74.1, 37.1 and 18.5 µmol/l), Tyr (218.3, 109.2, 54.6, 27.3 and 13.7 µmol/l) and Phe (240.0, 120.0, 60.0, 30.0 and 15.0 µmol/l) were prepared by diluting the stock solution with 0.1% formic acid. A mixed internal standard solution was made in 0.1% formic acid to concentrations of approximately 223.6, 70.8, 134.1, 54.0 and 73.4 µmol/l for Val-D_8_, Ile-D_10_, Leu-D_3_, Tyr-D_4_ and Phe-D_5_, respectively. The calibrators and the internal standard solution were stored in 1-ml aliquots at −80°C until the time of analysis.

#### Sample preparation

Calibrators, the internal standard and serum samples (if frozen) were thawed, mixed and equilibrated to room temperature. Aliquots of 0.05 ml of calibrators or serum samples were pipetted into 2-ml vials, followed by the addition of 0.05 ml of the internal standard solution and 0.4 ml of acetonitrile containing 0.1% formic acid to each vial. The vials were then vortexed and centrifuged at 3 148×g for 10 min. An aliquot of 0.05 ml of the supernatant was transferred to another vial and evaporated under a stream of nitrogen until dry. The residue was then reconstituted with 0.2 ml of 0.1% formic acid in water.

#### LC/MS/MS analysis

The LC separation was performed on an Agilent 1200 series LC system. Aliquots of 2 µl of the prepared samples were injected onto a Waters Shield C18 column (3.5 µm, 2.1×150 mm) maintained at 20°C and eluted with a mobile phase of 0.01% formic acid in water-acetonitrile (90∶10) at a flow rate of 0.3 ml/min. An API 4000 triple quadrupole mass spectrometer (Sciex Applied Biosystems) was used for the MS/MS detection. The detection was performed with positive electronic spray ionization (ESI) in multiple reaction monitoring (MRM) mode at a source temperature of 700°C and a voltage of 5500 V. The dwell times were 0.08 s for MRM. Nitrogen was used as the curtain, nebulizer and collision gas at pressures of 50, 60 and 70 psi, respectively. Certain ion transitions (see the Results section) for the amino acids and their internal standards were monitored and peak area ratios of amino acid to internal standard were calculated after correcting for transition overlaps of natural Leu and Ile (see the Results section). The calibration curve was generated using a linear regression of the peak area ratios (*y*) vs. the amino acid concentrations (*x*) of the calibrators.

### BCAAs and AAAs in Healthy Subjects

From a group of individuals attending an annual physical examination at the hospital, 192 subjects (94 males and 98 females ranging in age from 19 to 76 years) were selected. The subjects were deemed healthy on the basis of a medical history and physical examination results, with a body mass index (BMI) ranging from 16.6 to 29.9 and systolic blood pressure in the range of 90 to 140 mmHg. The sera from fasting blood samples obtained from volunteers were isolated and stored at −80°C until the time of analysis. The serum BCAAs and AAAs were analyzed by the present method. The serum samples were also tested for glucose, total cholesterol (TC), triglycerides (TG), high density lipoprotein cholesterol (HDL-C), low density lipoprotein cholesterol (LDL-C) and C-reactive protein (CRP). HDL and LDL subfraction cholesterol (HDL_2_-C, HDL_3_-C, LDL_a_-C and LDL_b_-C) levels were also measured for a subset of 111 samples. Glucose, TC, TG, HDL-C, LDL-C and CRP were measured using assay kits from Sekisui Medical Technologies (Osaka, Japan) on a Hitachi 7180 chemistry analyzer, and HDL and LDL subfractions were measured using our previously reported ultracentrifugation-HPLC method [18].

### Biological Variations of BCAAs and AAAs

Twenty laboratory professionals (10 males and 10 females in the age range of 23 to 58 years) at the hospital volunteered to participate in the study of biological variations of serum BCAAs and AAAs. The subjects maintained a constant weight and a normal diet and lifestyle during the study. Fasting blood samples were collected four times from each individual at two-week intervals, and the sera were separated and stored at −80°C. After completion of the sample collection, the samples were analyzed for BCAAs and AAAs with the presently described method. The intra-subject and inter-subject variations (CV_I_ and CV_G_) and the individuality index (II) were calculated as previously described by Fraser [19].

### Statistical Analysis

The present LC condition (mobile phase composition) was compared with another condition (lowered acetonitrile in the mobile phase) in the verification of the analytical specificity. The consistency of the two sets of results obtained under two LC conditions was evaluated by the paired student’s t-test, and numerical data were expressed as mean ± standard deviation (SD). The association of BCAAs and AAAs with other biochemistry parameters was assessed with the use of partial correlation coefficients after adjustment for age and gender. All reported *P* values are two-tailed, with a *P* value of 0.05 indicating statistical significance.

## Results

### LC/MS/MS Conditions and Specificity

**Figure 1:**
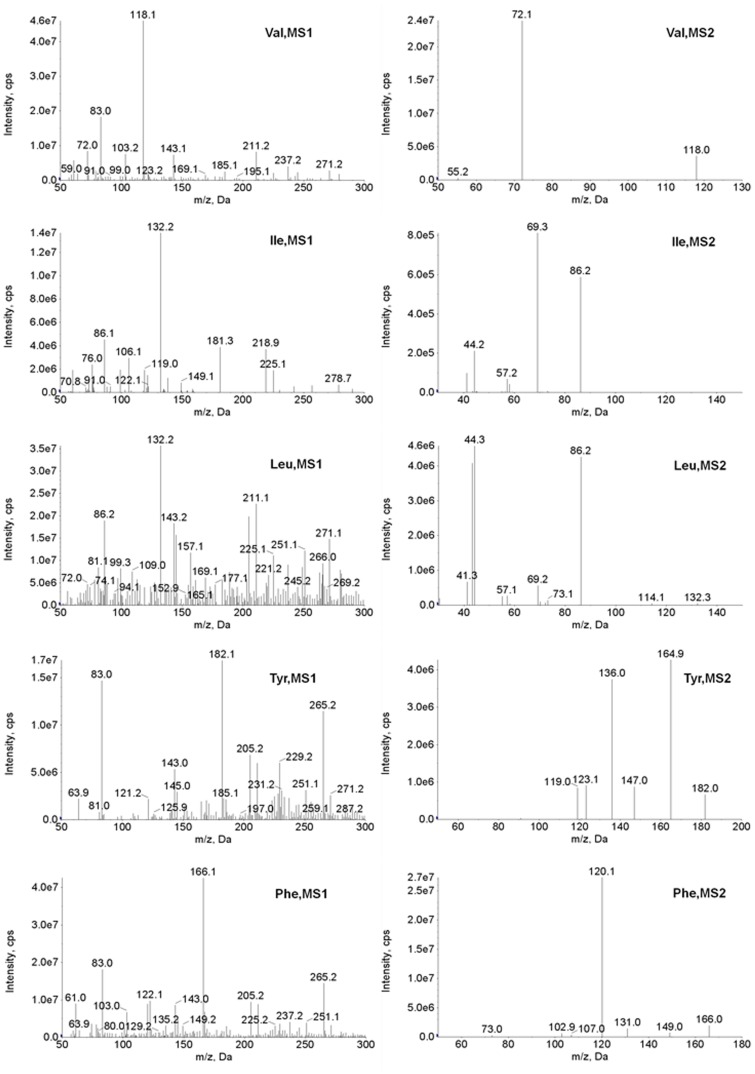
Ionization (MS 1) and fragmentation (MS 2) of each amino acid in tandem MS analysis. The data was collected as a 40 scan MCA (2 s/scan), i.e. each spectra is an accumulative result of 40 scans. The most abundant precursor and product ions for the amino acids were (M+H)^+^ and (M−COOH)^+^. The isobaric amino acids (e.g., Leu and Ile) have the similar and principal transition of *m/z* 132**→** *m/z* 86. The transitions of *m/z* 132**→** *m/z* 43 and *m/z* 132**→** *m/z* 69 were observed to be characteristic of Leu and Ile, respectively. It was also observed that the transitions were not specific, and two minor signals of *m/z* 132**→** *m/z* 69 and *m/z* 132**→** *m/z* 43 occurred for Leu and Ile, respectively. Val, valine; Ile, isoleucine; Leu, leucine; Tyr, tyrosine; Phe, phenylalanine; M, molecular weight; m/z, mass-to-charge ratio; MS, mass spectrometry.

**Figure 2:**
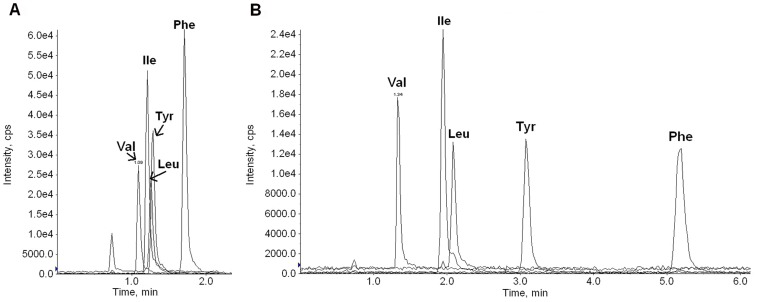
Multiple reaction monitoring (MRM) chromatograms of the five amino acids from a serum pool. The prepared sample was eluted with a mobile phase of **A)** 0.01% formic acid in water: acetonitrile (90∶10) or **B)** 0.01% formic acid in water: acetonitrile (98∶2). Val, valine; Ile, isoleucine; Leu, leucine; Tyr, tyrosine; Phe, phenylalanine.

**Figure 3:**
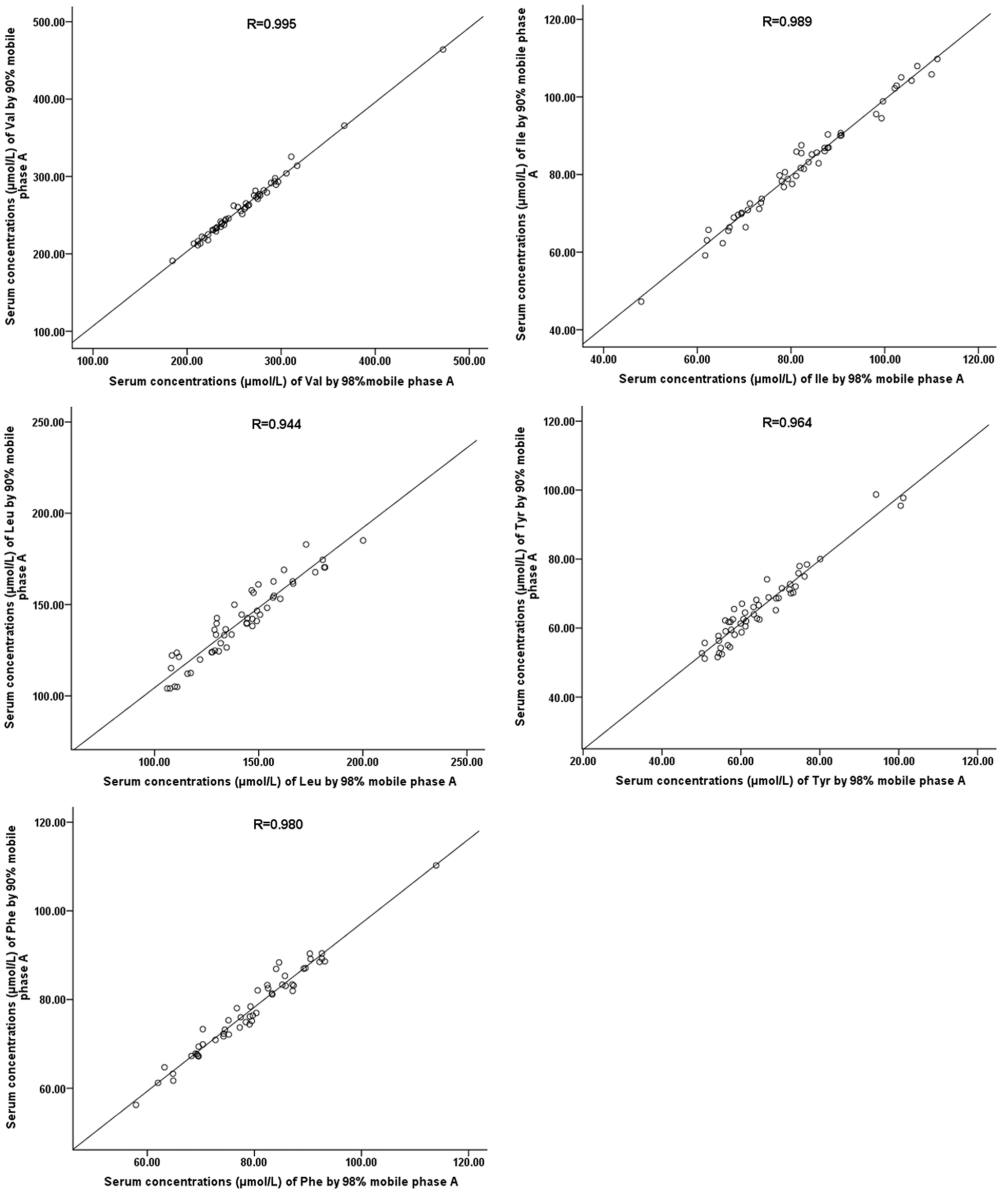
Correlation of the two sets of results from 51 serum samples under different mobile phase conditions. Mobile phase A: 0.01% formic acid in water. Mobile phase B: acetonitrile. Val, valine; Ile, isoleucine; Leu, leucine; Tyr, tyrosine; Phe, phenylalanine.

The ionization and fragmentation of the amino acids under various MS/MS conditions were tested by injecting each of the amino acids into the MS/MS system using a syringe pump. The MS/MS conditions were optimized for signal intensity and detection specificity. **Table 1** presents the MS/MS transitions and parameters used for monitoring the amino acids. The ionization and fragmentation of each amino acid under the selected conditions are shown in **Figure 1**. Generally, the most abundant precursor and product ions for the amino acids were (M+H)^+^ and (M−COOH)^+^; thus, the transition of (M+H)^+^ **→**(M−COOH)^+^ was used for the detection of Val, Tyr and Phe. For Leu and Ile, transitions of *m*/*z* 132**→** *m*/*z* 43 and *m*/*z* 132**→** *m*/*z* 69 were used, respectively, to separate the isobaric molecules. As shown in **Figure 1**, these two transitions were not specific to Leu and Ile, as a noticeable *m*/*z* 132**→** *m*/*z* 43 signal for Ile and a *m*/*z* 132**→** *m*/*z* 69 signal for Leu were also observed. Repeated analyses of Leu and Ile at different concentrations were performed, and consistent peak area ratios of 0.06 for *m*/*z* 132**→** *m*/*z* 69 to *m*/*z* 132**→** *m*/*z* 43 for Leu and 0.02 for *m*/*z* 132**→** *m*/*z* 43 to *m*/*z* 132**→** *m*/*z* 69 for Ile were observed. These ratios were then used to correct the peak areas of Leu (*A*_Leu, corrected_ = *A_m_*_/*z* 132**→***m*/*z* 43_–0.02×*A_m_*_/*z* 132**→***m*/*z* 69_) and Ile (*A*_Ile, corrected_ = *A_m_*_/*z* 132**→***m*/*z* 69_–0.06×*A_m_*_/*z* 132**→***m*/*z* 43_). Such corrections were not needed for their internal standards, because Leu-D3 and Ile-D10 (a mass difference of 7) were used as internal standards for Leu and Ile respectively. The use of the above transitions required minimum LC separation and enabled a run time of less than 2 min, as shown in **Figure 2A**. To verify the specificity of the analysis, 51 serum samples were analyzed with both the present chromatographic condition and a condition (lowered acetonitrile proportion in the mobile phase) that enabled separation of Leu and Ile, as shown in **Figure 2B**. The two sets of results and their differences are shown in **Figure 3** and **Table 2**. The results under the two conditions were generally comparable, though statistically significant differences were observed for Tyr and Phe.

**Table 1:** Ion transitions for the unlabeled and labeled amino acids (internal standards) and instrumental parameters for their MS/MS detection in MRM mode.

	Ion transition (m/z)	DP (psi)	EP (psi)	CE (psi)
Val	118.0 **→** 72.1	50	10	36
Val-D_8_	126.1 **→** 80.1	50	10	36
Ile	132.1 **→** 69.2	20	5	32
Ile-D_10_	142.1 **→** 78.1	20	5	32
Leu	132.1 **→** 43.1	20	5	36
Leu-D_3_	135.0 **→** 46.1	20	5	36
Tyr	182.0 **→** 136.0	50	10	30
Tyr-D_4_	186.0 **→** 140.0	50	10	30
Phe	166.0 **→** 120.1	50	6	40
Phe-D_5_	171.0 **→** 125.1	50	6	40

MS/MS, tandem mass spectrometry; MRM, multiple reaction monitoring; m/z, mass-to-charge ratio; DP, declustering potential; EP, entrance potential; CE, collision energy; Val, valine; Ile, isoleucine; Leu, leucine; Tyr, tyrosine; Phe, phenylalanine.

**Table 2:** Comparison of the two sets of results from 51 serum samples under different mobile phase conditions.

	Serum concentrations by Method 1 (90%Mobile phase A*) (Mean±SD) (umol/l)	Serum concentrations by Method 2 (98%Mobile phase A) (Mean±SD) (umol/l)	Averaged relativebias (%)	*P*
Val	260.8±44.1	259.7±45.5	0.5	0.09
Ile	81.9±13.9	82.1±14.0	−0.3	0.38
Leu	140.5±20.9	141.0±22.5	−0.2	0.54
Tyr	65.9±10.8	64.9±11.4	1.8	0.03
Phe	77.5±9.8	79.2±10.1	−2.1	<0.05

* Mobile phase A: 0.01% formic acid in water. Mobile phase B: acetonitrile.

The comparison was evaluated by the paired student’s t-test, Val, valine; Ile, isoleucine; Leu, leucine; Tyr, tyrosine; Phe, phenylalanine.

### Linearity

**Figure 4:**
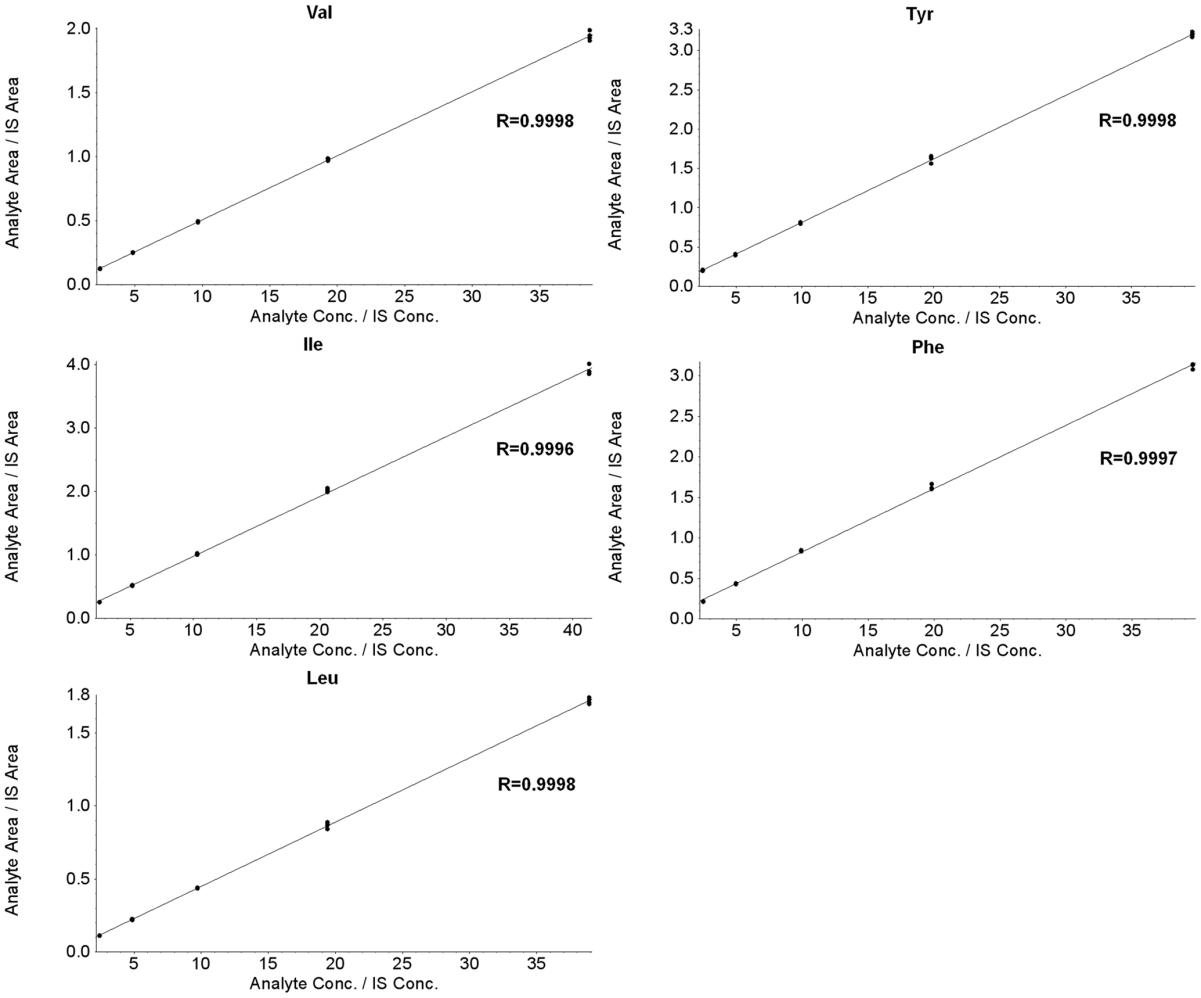
Representative calibration curves for each amino acid. Val, valine; Ile, isoleucine; Leu, leucine; Tyr, tyrosine; Phe, phenylalanine.

The linear regression between the peak area ratios and the amino acid concentrations was used for the establishment of the calibration function. Table 3 lists the average regression coefficients (*a*), intercept (*b*), correlation coefficient (*r* ^2^) and standard error of the estimate (SE) of the regression. Typical calibration curves for each amino acid are shown in **Figure 4**.

**Table 3:** Calibration parameters of serum BCAAs and AAAs by the LC/MS/MS method.

	Regression coefficients (*a*)	Intercept (*b*)	Standard error (SE) of the estimate (y)	Correlation coefficient (*r^2^*)
Val	0.05	0.02	0.01	0.9997
Ile	0.09	0.04	0.03	0.9996
Leu	0.04	0.01	0.01	0.9997
Tyr	0.08	0.02	0.02	0.9998
Phe	0.08	0.05	0.03	0.9995

BCAAs, branched-chain amino acids; AAAs, aromatic amino acids; LC/MS/MS, liquid chromatography tandem mass spectrometry; Val, valine; Ile, isoleucine; Leu, leucine; Tyr, tyrosine; Phe, phenylalanine.

### Recoveries

The recoveries of the analyses were tested by spiking a serum with known amounts of amino acids followed by analyzing the amino acids in the serum samples. Two levels of amino acids were added to the serum. As shown in **Table 4**, the average analytical recoveries for Val, Ile, Leu, Tyr and Phe were 100.9%, 103.2%, 101.8%, 103.4% and 101.8%, respectively. It should be noted that, while the analytical recoveries were close to 100%, the absolute recoveries of the amino acids were in the range of 60 to 77%, indicating the occurrence of ion suppression.

**Table 4:** Analytical recoveries of the LC/MS/MS method.

	Val	Ile	Leu	Tyr	Phe
Initial concentration(µmol/l)	241.6	66.4	131.7	63.4	82.9
*Spiked serum 1*					
Concentration (µmol/l)	326.0	147.9	207.5	120.0	144.0
Spiked standards (µmol/l)	82.5	78.7	74.1	54.6	60.0
Recovery (%)	102.3	103.6	102.4	103.6	101.9
*Spiked serum 2*					
Concentration (µmol/l)	406.0	228.3	281.90	176.2	204.9
Spiked standards (µmol/l)	165.1	157.4	148.2	109.2	120.0
Recovery (%)	99.6	102.8	101.3	103.3	101.7

LC/MS/MS, liquid chromatography tandem mass spectrometry; Val, valine; Ile, isoleucine; Leu, leucine; Tyr, tyrosine; Phe, phenylalanine.

### Precision, Sample Stability and Limits of Quantification

The precision of the analysis was tested by analyzing four serum pools of different amino acid concentrations. The serum pools were analyzed in triplicate in five independent runs. The intra-run and total *CV*s are presented in **Table 5**. Total *CV*s for the measurement of Val, Ile, Leu, Tyr and Phe ranged 1.4%–2.3%, 1.5%–2.1%, 1.8%–2.7%, 2.8%–3.2% and 1.4%–2.3%, respectively. The results for a quality control material in 15 runs within a period of 14 months showed similar total *CV*s (1.6%, 1.5%, 1.9%, 2.5% and 2.4% for Val, Ile, Leu, Tyr and Phe, respectively). The control material was a frozen serum pool and the above results also demonstrated that the serum amino acids were stable during the storage at −80°C. The limits of quantification (LOQ), defined as a concentration at which the signal-to-noise ratio is 10, were 0.3, 0.7, 2.6, 0.2 and 0.2 µmol/l for Val, Ile, Leu, Tyr and Phe, respectively.

**Table 5:** Precision of LC/MS/MS analysis of serum BCAAs and AAAs.

	Val	Ile	Leu	Tyr	Phe
*Serum 1*					
Mean (µmol/l)	228.5	68.1	129.9	56.4	88.0
Intra-run *CV* (%)	1.2	1.2	1.6	2.0	1.0
Total *CV* (%)	1.4	1.5	1.8	3.0	1.4
*Serum 2*					
Mean (µmol/l)	272.6	82.1	152.6	65.2	89.1
Intra-run *CV* (%)	1.8	1.4	2.0	1.9	1.8
Total *CV* (%)	2.3	2.1	2.7	3.2	2.3
*Serum 3*					
Mean (µmol/l)	269.1	93.9	158.0	73.6	91.6
Intra-run *CV* (%)	1.9	1.6	1.8	1.8	1.5
Total *CV* (%)	2.1	1.8	2.1	2.8	1.8
*Serum 4*					
Mean (µmol/l)	237.1	66.1	129.0	61.0	81.5
Intra-run *CV* (%)	1.6	1.4	1.4	2.0	1.5
Total *CV* (%)	2.0	1.8	2.1	3.0	1.8

BCAAs, branched-chain amino acids; AAAs, aromatic amino acids; LC/MS/MS, liquid chromatography tandem mass spectrometry; Val, valine; Ile, isoleucine; Leu, leucine; Tyr, tyrosine; Phe, phenylalanine; *CV*, Coefficient of Variation.

### BCAAs and AAAs in Healthy Subjects

Serum BCAAs and AAAs in 192 healthy subjects were analyzed. The distribution of BCAAs and AAAs in the subjects was leptokurtic and positively skewed. The mean and percentiles of the amino acids are listed in **Table 6**. Serum BCAAs in males were significantly higher than that in females (*P*<0.05), whereas AAAs were similar in the two groups. Serum BCAAs seemed to be negatively correlated with age, especially Ile (*P*<0.001). In partial correlation analyses adjusted for age and gender, as shown in **Table 7**, most of the BCAAs and AAAs were significantly correlated positively with BMI, glucose, TG, LDL_b_-C and CRP, and negatively correlated with HDL-C and HDL_2_-C. Serum AAAs also seemed positively correlated with blood pressure. No significant correlations between the amino acids and TC, LDL-C, LDL_a_-C or HDL_3_-C were observed.

**Table 6:** The concentrations of BCAAs and AAAs and percentiles (µmol/l) obtained by the LC/MS/MS method in 192 healthy subjects.

			Percentiles								
	Subset	Mean	2.5th	5th	10th	25th	50th	75th	90th	95th	97.5th
Val	all	251.5	184.5	194.3	207.1	222.9	244.9	275.4	298.6	321.4	354.0
	male	261.7	194.0	205.5	212.4	232.0	258.2	280.2	305.7	339.5	361.8
	female	241.7	183.7	186.8	200.0	220.0	232.5	260.3	291.2	299.9	359.4
Ile	all	73.7	50.6	54.0	56.8	62.7	70.5	83.3	95.6	100.3	107.7
	male	79.3	53.8	55.4	60.2	67.8	79.4	88.6	97.7	105.8	110.2
	female	68.4	48.4	52.1	55.5	59.4	65.6	72.6	84.4	99.1	102.8
Leu	all	136.7	103.9	106.6	109.9	121.4	134.1	150.2	165.9	178.0	188.9
	male	144.9	107.0	108.5	116.6	130.1	142.8	158.5	174.1	185.4	196.0
	female	128.8	97.9	104.1	106.8	116.3	126.0	138.8	154.2	165.4	174.3
Tyr	all	66.8	49.0	51.2	54.2	58.1	64.0	73.8	83.5	89.5	93.6
Phe	all	78.6	58.0	62.2	64.9	70.8	76.3	84.2	92.8	101.6	107.9

BCAAs, branched-chain amino acids; AAAs, aromatic amino acids; LC/MS/MS, liquid chromatography tandem mass spectrometry; Val, valine; Ile, isoleucine; Leu, leucine; Tyr, tyrosine; Phe, phenylalanine.

**Table 7:** Partial correlations (*r*) of BCAAs and AAAs with other parameters in 192 healthy subjects, adjusted for age and gender.

	Val	Ile	Leu	Tyr	Phe	BCAA	AAA
BMI	0.219**	0.213**	0.249***	0.254***	0.169*	0.236***	0.238***
SBP	0.043	0.015	0.023	0.198**	0.122	0.033	0.181*
DBP	0.074	0.072	0.095	0.189**	0.162*	0.083	0.198**
FBG	0.221**	0.176*	0.244**	0.218**	0.140	0.230**	0.205**
TC	0.122	0.065	0.111	0.042	−0.005	0.113	0.021
TG	0.322***	0.329***	0.341***	0.264***	0.231**	0.343***	0.279***
HDL-C	−0.231**	−0.282***	−0.267***	−0.254***	−0.267***	−0.262***	−0.294***
HDL_2_-C	−0.279**	−0.250**	−0.283**	−0.216*	−0.201**	−0.288**	−0.241*
HDL_3_-C	0.063	−0.114	0.005	−0.039	−0.094	0.014	−0.079
LDL-C	0.118	0.078	0.097	0.109	0.027	0.109	0.077
LDL_a_-C	0.051	0.061	0.078	−0.118	0.010	0.063	−0.059
LDL_b_-C	0.294**	0.181	0.164	0.355***	−0.048	0.248**	0.166
hs-CRP	0.119	0.141*	0.143*	0.122	0.124	0.136	0.139

* *P*<0.05;

** *P*<0.01;

*** *P*<0.001.

For lipoprotein subfractions, 111 cases were analyzed. BCAA represents the sum of concentrations of Val, Ile and Leu; AAA represents the sum of concentrations of Tyr and Phe; SBP, systolic blood pressure; DBP, diastolic blood pressure; FBG, fasting blood glucose; hs-CRP, high-sensitivity C-reactive protein; HDL_2_, high-density lipoprotein of d 1.063–1.125 kg/l; HDL_3_, high-density lipoprotein of d 1.125–1.210 kg/l; LDL_b_, small-dense LDL; LDL_a_, large-buoyant LDL. Val, valine; Ile, isoleucine; Leu, leucine; Tyr, tyrosine; Phe, phenylalanine.

### Biological Variations

Biological variations of serum BCAAs and AAAs were studied in 20 healthy volunteers. The CV_I_, CV_G_ and II for each of the amino acids are presented in **Table 8**.

**Table 8:** Intra-subject (CV_I_) and inter subject (CV_G_) biological variations of BCAAs and AAAs.

	Median (range)(µmol/l)	CV_I_ (%)	CV_I_ range(%)	CV_G_ (%)	II
Val	244.0 (157.2∼336.8)	9.8	1.2∼30.4	13.1	0.8
Ile	74.1 (51.8∼116.6)	11.7	1.4∼33.0	19.4	0.6
Leu	132.9 (89.8∼176.4)	9.5	2.4∼26.2	14.8	0.6
Tyr	66.7 (45.9∼98.6)	9.3	0.8∼18.7	16.2	0.6
Phe	72.1 (52.5∼109.3)	8.3	0.5∼24.2	17.1	0.5

BCAAs, branched-chain amino acids; AAAs, aromatic amino acids; Val, valine; Ile, isoleucine; Leu, leucine; Tyr, tyrosine; Phe, phenylalanine.

## Discussion

It has long been observed that BCAAs and AAAs correlate with obesity and serum insulin [20], but only recently have these amino acids been found to be closely associated with insulin resistance and pathogenesis of diabetes [21], [22]. Several recent studies have consistently shown that BCAAs and AAAs are strongly correlated with insulin resistance [2]–[4], prediction of diabetes development [5], [6] and intervention outcomes [23]–[25] and are uniquely responsive to therapeutic interventions. All of these findings suggest that serum BCAAs and AAAs be promising early markers of diabetes development. Further studies are warranted to test whether these amino acids might help identify candidates for interventions to reduce diabetes risk; therefore, convenient methods for measuring the amino acids are needed.

Among the various analytical techniques for amino acids, LC/MS/MS, which involves an LC separation and a combined MS analysis of a precursor ion and one of its fragments, has the minimum requirements for sample preparation and chromatographic separation. However, direct analysis of amino acids by LC/MS/MS can be challenging due to the existence of isobaric amino acids (e.g., Leu and Ile) that have similar LC and MS properties. Most of the current LC/MS/MS methods [16], [17] use the principal transition of Leu and Ile (*m/z* 132**→** *m/z* 86) and separate the amino acids by LC. Understandably, the LC separation often requires a relatively long chromatography time.

There are other MS transitions for Leu and Ile that might be useful for the detection of amino acids [13], [14]. In trying to differentiate Leu and Ile by MS, we tested the fragmentations of the two molecules under various MS conditions. The transitions of *m/z* 132**→** *m/z* 43 and *m/z* 132**→** *m/z* 69 were observed to be characteristic of Leu and Ile, respectively, and their abundances were sufficient for the detection of the amino acids in serum under certain MS conditions. It was also observed, however, that the transitions were not specific, and two minor signals of *m/z* 132**→** *m/z* 69 and *m/z* 132**→** *m/z* 43 occurred for Leu and Ile, respectively. If the relative magnitude of the interfering transitions was known and consistent, the observed transitions could be corrected and used for the detection of Leu and Ile. Thus, we then analyzed pure Leu and Ile at different concentrations and obtained consistent peak area ratios (6% and 2% with *CV*s of 12% and 16%, respectively) of *m/z* 132**→** *m/z* 69 to *m/z* 132**→** *m/z* 43 for Leu and *m/z* 132**→** *m/z* 43 to *m/z* 132**→** *m/z* 69 for Ile. These ratios were then used for the correction of the peak areas in the present method. For simplicity, an approximate direct correction was used as described in the Results section. The approximation causes negligible errors (∼0.1%) because of the low intensity of the interferences. Furthermore, the corrections enabled the use of the transitions for specific detection of Leu and Ile, and thus a short LC time.

There are other substances that are isobaric to Leu and Ile and that may also be present in serum [15], [17], [26]. Allo-isoleucine, a marker of maple syrup urine disease, and 6-aminocaproic acid, a therapeutic drug, exhibit the transition of *m/z* 132**→** *m/z* 69. Although this may potentially interfere with the Ile analysis, these substances are essentially absent in normal serum. Hydroxyproline, which is normally present in serum, was tested for its LC and MS/MS properties. Although a weak *m/z* 132**→** *m/z* 69 transition was observed, it showed a much shorter retention time and did not interfere with the analysis.

Based on previous studies [15], [17], there should be no other substances that interfere with the present BCAA and AAA analysis. To verify the specificity of the method, a set of 51 serum samples were analyzed with two different LC conditions, the results of which were also compared.

Ion suppression would be expected for an LC/MS/MS method with limited sample preparation and a short LC time. Indeed, ion suppression at a magnitude of 20–40% was observed for the amino acids; however, the effect was largely corrected by the use of isotopically labeled internal standards and would have little influence on the accuracy of the method, as indicated by the analytical recoveries (99.6–103.6%).

The use of weaker transitions for Leu and Ile and the occurrence of ion suppression, naturally reduce the analytical sensitivity of the method. However, the amino acids are relatively abundant in serum, and the LOQs of the method were well below the normal serum levels. Other validation or assessment studies showed the method had sufficient linearity and precision for the measurement of serum BCAAs and AAAs.

As serum BCAAs and AAAs have emerged as potential early diabetes risk factors, it is of interest to look at the distribution of the amino acids and their correlation with known risk factors in apparently healthy subjects. With this method, we measured serum BCAAs and AAAs in 192 healthy subjects. Preliminary reference intervals were established for the amino acids as shown in **Table 6**. More interestingly, it was observed that serum BCAAs and AAAs were very closely correlated to most of the risk factors for diabetes and cardiovascular diseases (**Table 7**).

Biological variations (CV_I_, CV_G_ and II) are important characteristics of clinical laboratory tests that help not only the use and interpretation of the tests but also the definition of analytical goals. Biological variations of serum BCAAs and AAAs were also studied as shown in **Table 8**.

In conclusion, we have developed a simple, fast and reliable ID-LC/MS/MS method to directly determine BCAAs and AAAs in human serum. This method may serve as a convenient tool in the study of diabetes risks.
